# Quadratic few-cycle solitons on chip

**DOI:** 10.1038/s41377-026-02312-7

**Published:** 2026-05-08

**Authors:** A. V. Ermolaev, L. Emonin, J. M. Dudley

**Affiliations:** 1 Université Marie et Louis Pasteur, CNRS Institut FEMTO-ST, 25000 Besançon, France; 2 Institut Universitaire de France, 75231 Paris, France

**Keywords:** Nanocavities, Ultrafast photonics

## Abstract

Experiments in a dispersion-engineered nanophotonic lithium niobate waveguide have demonstrated two-color soliton compression to few-cycle duration by exploiting cascaded second-order nonlinearity. These results open new opportunities to study quadratic soliton dynamics in integrated platforms and for applications in on-chip photonic signal processing.

Solitons in optical fiber are amongst the most remarkable nonlinear structures in optics, with stability and robustness that arises due to the balance between linear dispersion and self-phase modulation from the third-order (cubic) χ^(3)^ Kerr nonlinearity^1^. There has been intense interest in both their fundamental characteristics as well as their many applications, shaping much of modern ultrafast optics, including long-haul transmission, femtosecond laser design and supercontinuum development^2–4^.

Yet it has also been known since the first decades of nonlinear optics that soliton localization can arise from the cascaded second-order χ^(2)^:χ^(2)^ nonlinearity, where dispersion is balanced through the nonlinear phase shift developing as two fields of different frequency interact through parametric nonlinear coupling^5–9^. In fact, for the specific case of a strongly phase-mismatched interaction, it is possible to map these coupled wave interactions onto a nonlinear Schrödinger equation, such that these *quadratic soliton* dynamics emulate that seen in a fiber system^10^. Whilst this equivalence has certainly been extremely valuable, it has had the unintended consequence that the rich physics of quadratic solitons beyond the Kerr-equivalent regime has remained relatively unexplored. In a sense, this is somewhat surprising because the quadratic nonlinearity is known to possess numerous potential advantages, such as a stronger inherent nonlinear strength and the possibility to tune the sign of the phase mismatch to excite soliton dynamics in both anomalous and normal group velocity dispersion regimes. However, many of the previous experiments on quadratic solitons have suffered from group velocity walk-off between the interacting two-color fields, limiting the interaction length over which coupled soliton propagation can be observed^11^.

It is here where the recent work of Gray et al. has made a major contribution in overcoming this limitation to show how the landscape of cascaded second-order nonlinearity can be more generally accessed and exploited^12^. In particular, using careful design of a nanophotonic lithium niobate waveguide (optimizing both dispersion and nonlinear characteristics), they have been able to mitigate the effect of walk-off to directly observe pulse compression to only a few optical cycles. Of particular significance is that these experiments have been carried out far from the large phase-mismatch limit so that the soliton dynamics are seen in a regime in which the quadratic interaction cannot be reduced to an effective Kerr nonlinearity. This is a major novelty in showing how a much wider range of two-color soliton dynamics in χ^(2)^ media can be readily accessed in the laboratory, and it opens up exciting possibilities for ultrafast optical signal processing with new degrees of freedom.

Their experimental system used nearly transform-limited ~35 fs FWHM pulses at 2090 nm coupled into a 6.5 mm long thin-film lithium niobate nanophotonic waveguide to generate a two-color soliton state between the 2090 nm fundamental and the 1045 nm second harmonic. The waveguide design and length were chosen for optimal soliton compression, guided by both analytic soliton theory and numerical simulations of the coupled wave equations. For an on-chip fundamental pulse energy of 3 pJ, they observed 13 fs FWHM pulses at the fundamental and 17 fs FWHM pulses at the second harmonic. These measurements together with observed strong back-and-forth energy exchange directly reveal the signatures of genuine quadratic soliton dynamics in a waveguide platform. The ability to perform these measurements on the pure temporal dynamics in a waveguide platform is extremely important for comparison with quadratic soliton theory, as previous experiments in bulk media have often involved spatio-temporal interactions that have made unambiguous interpretation of the results difficult.

Beyond its evident conceptual importance, this work has immediate implications for ultrafast pulse compression and nonlinear pulse shaping in integrated optics, and Table 1 positions the results relative to a selection (non-exhaustive) of other studies in the field^13–22^. Their results also draw attention to the importance of waveguide design to optimize dispersion and nonlinearity for specific applications, particularly in view of recent advances and availability of nonlinear materials^23^.

**Table 1:** A selection of pulse-compression demonstrations across χ^(2)^ and χ^(3)^ platforms

Material & Platform	Nonlinearity	Length	Compressed duration & central wavelength		Input energy
Hollow-core fiber & chirped mirrors^13^	Kerr	6 m	5.1 fs	1030 nm	1 mJ
Gas-filled stretched hollow-core fiber^14^	Kerr	3 m	1.2 fs	800 nm	337 μJ
SiN waveguide with SiO_2_ cladding^15^	Kerr	40 cm	66 fs	1540 nm	25 pJ
USRN waveguide^16^	Kerr	7 mm	230 fs	1550 nm	~16 μJ
USRN incorporated in silica with grating^17^	Kerr	6 mm	550 fs	1553.5 nm	58 pJ
GaInP PC membrane^18^	Kerr	1 mm	900 fs	1551 nm	20 pJ
			1100 fs	1555 nm	
Silicon PC air-suspended^19^	Kerr	0.4 mm	1600 fs	1547 nm	9 pJ
			57 fs	1570 nm	
Aperiodically-poled MgO:LiNbO_3_ (bulk)^20^	Quadratic	50 mm	54 fs	785 nm	~3 μJ
BBO (bulk, 3 stage)^21^	Quadratic	26 mm	30 fs	1030 nm	4.2 μJ
Lithium-niobate crystal (bulk)^22^	Quadratic	1 mm	17 fs	1300 nm	~200 μJ
MgO-doped thin-film lithium niobate on a SiO_2_/Si substrate^12^	Quadratic	6.5 mm	13 fs	2090 nm	3 pJ
			17 fs	1045 nm	

*PC* Photonic Crystal, *USRN* ultra-silicon-rich nitride

More broadly, these results arrive at a time when there is intense interest in developing waveguide platforms that reproduce and extend nonlinear and dispersive phenomena that have been previously seen in optical fibers. Integrated photonic systems are now routinely engineered for applications in ultrafast optics^24–32^, and within this rapidly evolving field, the demonstration of quadratic solitons beyond the Kerr-equivalent regime is likely to stimulate much new work studying soliton dynamics in χ^(2)^ waveguide platforms.
